# Digital Addiction and Sleep

**DOI:** 10.3390/ijerph19116910

**Published:** 2022-06-05

**Authors:** Birgitta Dresp-Langley, Axel Hutt

**Affiliations:** 1 Centre National de la Recherche Scientifique, UMR7357 CNRS, ICube Research Department, Université de Strasbourg and Hôpitaux Universitaires de Strasbourg, Pavillon Clovis Vincent, F-67085 Strasbourg, France; 2 Team MIMESIS, INRIA, UMR7357 CNRS, ICube Research Department, F-67085 Strasbourg, France; axel.hutt@inria.fr

**Keywords:** digital environments, overexposure, internet, addiction, iRISA syndrome, melatonin, vitamin D, sleep loss, depression, obesity, serotonin, dopamine, oxidative stress

## Abstract

In 2020, the World Health Organization formally recognized addiction to digital technology (connected devices) as a worldwide problem, where excessive online activity and internet use lead to inability to manage time, energy, and attention during daytime and produce disturbed sleep patterns or insomnia during nighttime. Recent studies have shown that the problem has increased in magnitude worldwide during the COVID-19 pandemic. The extent to which dysfunctional sleep is a consequence of altered motivation, memory function, mood, diet, and other lifestyle variables or results from excess of blue-light exposure when looking at digital device screens for long hours at day and night is one of many still unresolved questions. This article offers a narrative overview of some of the most recent literature on this topic. The analysis provided offers a conceptual basis for understanding digital addiction as one of the major reasons why people, and adolescents in particular, sleep less and less well in the digital age. It discusses definitions as well as mechanistic model accounts in context. Digital addiction is identified as functionally equivalent to all addictions, characterized by the compulsive, habitual, and uncontrolled use of digital devices and an excessively repeated engagement in a particular online behavior. Once the urge to be online has become uncontrollable, it is always accompanied by severe sleep loss, emotional distress, depression, and memory dysfunction. In extreme cases, it may lead to suicide. The syndrome has been linked to the known chronic effects of all drugs, producing disturbances in cellular and molecular mechanisms of the GABAergic and glutamatergic neurotransmitter systems. Dopamine and serotonin synaptic plasticity, essential for impulse control, memory, and sleep function, are measurably altered. The full spectrum of behavioral symptoms in digital addicts include eating disorders and withdrawal from outdoor and social life. Evidence pointing towards dysfunctional melatonin and vitamin D metabolism in digital addicts should be taken into account for carving out perspectives for treatment. The conclusions offer a holistic account for digital addiction, where sleep deficit is one of the key factors.

## 1. Introduction

The notion of “death by digital distraction” as the extreme consequence of personal technology use first appeared on the internet a few years ago in an alarming publication by the U.S. Naval Institute [1]. The publication explicitly considers digital technology as a powerful “new drug”, referring to national statistics suggesting that 78% of Americans between 18 and 24 years of age use social media, online dating, video games, online pornography, and other personal digital technology multiple times a day [2], often to an extent that raises grounds for concern. Among specific problems mentioned occurring in young Navy recruits is that boys are becoming increasingly preoccupied with video games and girls more interested in social media activities than in direct interaction with other individuals, and there is also a recent and steep increase in symptoms indicative of technology addiction [3,4,5,6,7,8], such as depression, anxiety, loneliness, withdrawal from friends and family, sleep deprivation, and mood disorders. Statistical analyses [1,7,8] point towards a measurable increase in incidents, accidents, and failures during missions the nonstop comparison of one’s own with others’ digital “lives” as a source of depression, with suicide as the potentially ultimate escape from the many harmful effects of digital technology abuse in young people aged between 15 and 30. The year in which the U.S. Navy released their proceedings on technology addiction [1], the World Health Organization [9] formally recognized technology addiction as a worldwide problem, pointing out that addictive internet use leads to inability to manage and balance time, energy, and attention. Sleep, motivation, memory, eating habits, mood, social interactions, and lifestyle patterns are affected interdependently, and as a result, the psychological functioning and well-being of digital addicts is holistically jeopardized [4,5,6,7,8]. Daily online experience may seem harmless to the individual user; yet, it has potentially dramatic consequences when repeated compulsively, as it triggers neurochemical processes that kick off the brain’s reward circuitry in the same way as substances such as alcohol, nicotine, or cocaine [7]. Equivalent reward mechanisms govern addictive responses to sex, gambling, or driving a fast car at high speed [3]. The narrative review that follows is to convey a holistic understanding of the implications of digital addiction for lifestyle, sleep, and mental health of individuals, with a conceptual focus on underlying brain-behavior dynamics. The review strategy here builds upon bibliographic research of the international medical science database *MEDLINE* using *PubMed* hosted by the NIH. Keywords for the search include (internet addiction), yielding 89 papers taken into account; (internet addiction) + (sleep), yielding 26 papers taken into account; and (internet addiction) + (brain), yielding 11 papers taken into account. Additional keywords yielding further *MEDLINE* references include (behavioral addiction) + (reward) and (blue light exposure) + (health). In total, 176 documents of the 184 cited here originate from the *MEDLINE* search. The eight remaining documents cited here include public reports by the U.S. Navy, the World Health Organization, and the European Commission. These documents are available online.

## 2. Digital Addiction: A New Problem Worldwide

The first cell phone was released on the market in the USA in 1973, and about forty years later, more than half the U.S. adult population was estimated to be carrying a cell phone. In 2018, 77% of the USA population officially owned a smartphone, and that proportion is now approaching 95% [5]. Internet addiction [6] is a disabling condition that calls for full consideration worldwide. Reported to have a severe impact on young people’s brain function, internet addiction disorder (IAD) may also be referred to in terms of pathologic or problematic internet use (PIU) and is widely defined in terms of an impulse control disorder characterized by uncontrolled internet use [6,7]. A sub-category of IAD, internet gaming disorder (IGD), which specifically concerns videogame addiction [8], is included in Section 3 of the DSM 5. It is currently envisaged to include IAD and IGD also in the ICD-11, the World Health Organization’s [9] International Classification of Diseases for mortality and morbidity statistics. Since 2013, the American Psychiatric Association has included a specific form of digital addiction, internet gaming disorder, in the appendix of the updated version of the Diagnostic and Statistical Manual for Mental Disorders (DSM-5) [10]. Sleep problems are included therein as the third of nine criteria that are the keys to identifying IGD as defined by DSM-5: (1) playing games for long periods, (2) skipping school more often as a consequence, (3) experience sleep problems as a consequence, (4) feeling addicted to gaming, (5) resorting to gaming to escape adverse moods, (6) being preoccupied by gaming, (7) giving up other activities to gaming, (8) diminished social tolerance, and (9) withdrawal from social interactions as a consequence. Global trend analyses [11] point towards factors such as overall internet penetration per country and estimated internet use *per capita* as the ground on which digital addiction has grown over the last decades.

### 2.1. Instant Availability of the “Digital Drug”

In digital addiction, the internet is a channel [12] through which individuals may access whatever content they want (games, social media, shopping, sex, and so forth), wherever they want, and whenever they want it. The development of the addictive response is thereby digitally facilitated and instantly available to anyone—adults, adolescents, and young children [13]—through a smartphone, tablet, laptop, or computer workstation, with no intermediate dealer needed, as is the case with other types of addiction to substances or specific activities not performed online. At an advanced stage, IAD is associated with a significant and permanent symptomatic state at the psychological, cognitive, and physiological level, engendering more or less severe general functional impairment [14,15,16]. Reported symptoms include clinically measurable psychological stress [17,18,19,20], anxiety and depression [21,22,23], eating disorders [24,25,26], sleeplessness [27,28], and mood changes with suicidal ideation [29]. Cross-national studies on IAD involving more than 89,000 participants from 31 nations [15] suggest a global prevalence estimate for IAD of 6% worldwide. The highest estimates for IAD prevalence were reported for the Middle East for about 12% of the reference population, and the lowest prevalence was suggested for Northern and Western Europe with about 2.5% of the reference population. Comparison of national statistics for internet penetration or time spent online [13] do not suffice to explain such differences between countries or cultures [13]. A multitude of interdependent variables [3,6,7,8], such as socio-cultural factors (demographic variables; access to and acceptance of the internet), biological vulnerabilities (genetic predisposition, pre-existing metabolic disorders), and psychological factors (personality characteristics, negative affect) play a role here. Complex interactions between environmental, metabolic, and neurobiological changes in the brain, developed further below, would need to be taken into account. While internet addiction quite clearly has become a universal issue, the reported estimates from the different currently available publications and statistics vary considerably between countries. Two factors have been considered to explain the cross-national variations. One is internet access [9], which varies between continents and nations and predicts that IAD prevalence should be positively related to the internet penetration rate in a given country. The other factor is real-life quality [15,16], predicting that IAD prevalence should be inversely related to the global national index of life satisfaction and/or other specific national indices of environmental and lifestyle quality. However, whether progressing IAD yields lower life quality or whether low life quality promotes the onset of IAD in the first place is an open question. Studies on adolescents and college students [26,30] have identified a gender bias revealing continuous online availability and privileged use of the internet for seeking new friendships or relationships as risk factors in male students. Concomitantly, higher computer skills and ready internet access were identified as leading to higher risk for developing IAD. What is known is that IAD is significantly correlated with insomnia and depression. The varying levels of depression and insomnia related to IAD and more specifically online social networking addiction have been assessed using the Center for Epidemiological Studies Depression Scale [31], the Pittsburgh Sleep Quality Index [32], Young’s Diagnostic Questionnaire for Internet Addiction or Internet Addiction Test [33], and the Social Networking Addiction Scale [32,34] in cross-national studies [15,23,27,30]. The results show beyond reasonable doubt that high prevalence of IAD and online social networking addiction in any given population is described by total loss of impulse control associated with increased risks of developing insomnia and depression. Digitally mediated activities thus procure users with what, in [1], is referred to as the “new drug” and what we will refer to here as the digital drug. Repeated compulsory behavior, loss of impulse control, and, ultimately, digital addiction describe a complex syndrome that is triggered, facilitated, and maintained by the instant availability of the craved contents through the digital medium.

### 2.2. From Loss of Impulse Control to Insomnia and Depression

Depression in digital addicts is related to sleep disorders and insomnia associated with the addictive behavior [20,35]. Insomnia may be both a trigger and booster and a chronically developing consequence of the addiction. It can therefore be deemed adequate to consider addiction to internet activities, insomnia, and depression as a whole syndrome. Higher incidence of depression, insomnia, and suicide rates in teenagers [29] as a direct consequence of internet addiction are recognized as sources of worldwide concern [9]. Current research [14,27,29,36] points towards altered punishment-reward sensitivity and deregulation of the dopamine transmitter pathways in the brain as physiological correlate of digital addiction. This supports the postulate that it is equivalent to any other form of addiction although some still claim that digital addiction does not exist or is not understood well enough [37,38,39,40]. For almost a decade now, experts express consensus that all entities capable of stimulating a person can be addictive. Whenever a habit changes into a compulsory and repetitive pattern of behavior that can no longer be controlled by the individual without help from outside, the habit can be considered an addiction [41]. Research on the etiology and particular symptoms of digital addiction [6,7], by comparison with other behavioral addictions [3], is focused on sleep and mood disorders and recognizes that there are clear similarities in this regard between substance or behavioral addictions [3], including digital addiction [7]. A key symptom common to all forms of addiction is, indeed, sleep dysfunction [42,43].

### 2.3. Dysfunctional Sleep as a Key Variable

Sleep quality is, together with a balanced diet and regular exercise, one of the major conditions for good health. The annual costs of insomnia worldwide are estimated in hundreds of billions USD. Such estimates include statistics relative to people who suffer injuries every year due to sleep-related accidents and those who die because of sleep-related accidents. Individuals with frequent sleep disturbances report missing work and other important events and making errors at work and while driving [44,45]. Preserving well-regulated circadian rhythms is known to lower the risk of sleep disorders, psychological problems, and chronic health issues, such as eating disorders, obesity, and diabetes. Circadian rhythms, sometimes also called sleep–wake cycles [46], refer to a cyclic metabolism governed by factors within the body under the regulating influence of environmental factors such as sunlight, food quality, and regular exercise to help the body maintain them [47]. Digital addiction is a syndrome described by the compulsive need to spend an unreasonable amount of time, at day and at night, on the internet, to a point where healthy eating, outdoor activities with daylight exposure, relationships, work, exercise, and sleep are severely compromised. It is hardly surprising that this has an important influence on sleep–wake cycles, leading to sleeplessness and other sleep disorders, including insomnia [27,48]. Over the last decade, the prevalence of internet abuse among adolescents has risen steeply. In the U.S. and Japan, for example, 93% of adolescents between twelve and seventeen years old go online for several hours in the day and often also at night [49,50]. In India and China, the estimates range between 70% and 75% [20,51,52]. About 65% of internet addicts have reportedly higher incidence of psychological problems and mood disorders, and about 47% of them report repeated suicidal thoughts (suicidal ideation) in a week, and about 23% of digital addicts report at least one suicide attempt, and some of them report a history of several attempts in one year. Internet addiction and problematic internet use behavior always influence the sleep–wake cycle, leading to sleeplessness [53,54]. Extreme internet abuse is systematically associated with insomnia [55]. On the one hand, excessive day and night-time computer use causes a state of almost permanent arousal and blocks the calming effects of relaxation that are essential for preparing the body for good sleep [56,57]. On the other hand, sleep deprivation and depression are well known to be mutually reinforcing [56,57,58], and the complex interplay between depression and sleep disorder may engender the deregulation of circadian rhythms by reinforcing negative moods and by decreasing regular exposure to daylight, healthy exercise, eating patterns, and mood-regulated social activities [58]. In China in particular but also in Turkey, problematic internet use was significantly linked to depressive signs and sleep disorders [15,59,60]. In randomly sampled students from fifteen schools in Belgium [61], the children who spent more time on the internet went to bed significantly later during the week and also on weekends. They reportedly woke up later on weekends, spent less time in bed in the week, and experienced higher levels of tiredness. In high school students from a study in South Korea [62], the odds of excessive daytime drowsiness were significantly higher in internet addicts compared with non-addicts. Other frequent symptoms of digital addiction and problematic internet use [55,63,64] include migraines, backache, eating disorders, obesity, behavioral and emotional problems, and social withdrawal. The impact of electronic media use on sleep in school-aged children and adolescents is reflected by several variables reviewed in [65] and investigated across studies. Such are delayed bed time (DBT), total sleep time (TST), sleep onset latency (SOL), wake after sleep onset (WASO), or sleep efficiency (SE), with delayed bedtime (DBT) and shorter TST as the most consistently related to media use [65]. Dysfunctional sleep may be a consequence or the root cause of all these symptoms [57,64]. Two years ago, the emergence of the COVID-19 pandemic has since affected the lives of many people, including adolescents and young students. A cross-national study [66] explored internet addiction and changes in sleep patterns among medical students during the pandemic, assessing the relationship between these two variables. The cross-sectional study was carried out in seven countries, including the Dominican Republic, Egypt, Guyana, India, Mexico, Pakistan, and Sudan, using a convenience sampling technique and an online survey comprising demographic details and information regarding COVID-19. Scores from the Pittsburgh Sleep Quality Index (PSQI) [30] and Young’s Internet Addiction Test (IAT) [31] were used. Of a total of 2749 participants, 67.6% scored above 30 in the IAT, suggesting the presence of an internet addiction, and 73.5% scored equal to and above 5 in the PSQI, suggesting poor sleep quality. Internet addiction was found to be significant predictors of poor sleep quality, causing 13.2% of the variance in poor sleep quality. Participants who reported COVID-19-related symptoms had disturbed sleep and higher internet addiction levels when compared with those who did not. Participants who reported a diagnosis of COVID-19 reported poor sleep quality. Those living with a COVID-19-diagnosed patient reported higher internet addiction and worse sleep quality compared with those who did not have any COVID-19 patients in their surroundings. These results reveal the considerable impact of pandemic related stresses on digital addiction and sleep quality on students. To further understand the many factors involved here and their interdependency, we need insight into what happens in the brains of individuals addicted to online activities.

## 3. What Happens in the Brain?

What happens in the brain of digital addicts can be assimilated to what happens in any form of addiction. Previously [67], addictive behavior has been operationally defined as any behavior that features six core components of addiction: salience, mood modification, tolerance, withdrawal symptoms, conflict, and relapse. Any behavior that fulfils all these six criteria can be considered an addiction. Earlier, [68] defined addiction as a chronic, relapsing brain disease that results from prolonged effects of a drug on the brain, leading to compulsive consumption and abuse. Initial drug intakes are associated with the pleasure and positive reinforcement they produce through increased dopaminergic transmission in the mesocorticolimbic brain circuitry [69], which interacts with other brain circuits involved in executive functions. These functions concern inhibitory control related to decision making, attribution of added value, conditioning, memory, habitual responses, reward, motivation [70], reactivity to stress, energy, mood, hedonic states, and awareness of internal disturbances and changes within the body [71,72].

### 3.1. From Habit and Reward to Craving

The neural circuitry for reward has evolved across the phylogenic scale as the brain substrate of behavioral adaptation [73]. Dopamine plays a critical role in this circuitry, for the subjective pleasure associated with positive rewards, and the motivation or drive-related reinforcements associated with eating, drinking, or drugs [73,74]. Dopamine thus governs the reinforcement of normal as well as pathological response habits by modulating synaptic plasticity [75], a central factor in neuroadaptation theories of addiction [76]. In long-term addicts, the loss of control over consumption produces a brain state where the expression of dopamine receptors is decreased. As a consequence, activities that are not already “stamped in” by habitual reward are suppressed [71]. Loss of control results from a combination of repeated consumption to relieve the negative effects of withdrawal, compulsion to take the drug despite its negative effects, and craving, which precipitates relapse. Loss of control and the major symptoms associated with it have been functionally related to disruptive processes in the neural networks of the prefrontal cortex, i.e., alterations in network-specific functional connectivity, producing the core clinical symptoms of drug addiction, the **iRISA** (**i**mpaired **R**esponse **I**nhibition and **S**alience **A**ttribution) syndrome [76]. At the social level, the loss of control may produce a downward spiral of progressive disinterest in anything else than the drug, alexithymia, and suicidal ideation [77]. In digital addiction, instead of coping or learning to cope with problems through real-world interaction with other human beings, the subject consumes the digital drug in the same craving way. Like other addicts, the individuals progressively spiral down a slippery slope of progressive isolation, insomnia, and depression [15,21,76,77,78]. The initially pleasant, so-called rewarding effects of the drug are relayed by the release of dopamine in the nucleus accumbens (NA) by the synaptic endings from the neurons of the ventral tegmental area (VTA) of the mesocorticolimbic circuitry [79,80]. The release of dopamine in the NA plays a major role in the development of addiction, as this brain structure constitutes a “crossroads system” towards which different transmission routes from various brain structures such as the amygdala, the frontal cortex, and the hippocampus converge, driving motivated action through connectivity with the extrapyramidal motor system [81,82,83]. Besides the mesolimbic circuit (VTA and NA), other dopaminergic pathways contribute to the rewarding effects of drugs and addiction, such as the mesostriatal pathways with dopaminergic neurons of the substantia nigra projecting into the dorsal striatum [81,82,83], and the mesocortical pathways with dopaminergic neurons of the substantia nigra projecting into the frontal cortex [83]. Projection-specific intrinsic characteristics and the differential afferent inputs onto these VTA neuron subpopulations consolidate findings of drug-induced plasticity of VTA neurons and highlight the importance of future projection-based studies of this system to understand what happens in the brain in the progression from initial pleasure to dependence [84]. This involves (1) automatisms in which the initially motivated behavior subsequently becomes a habit and (2) a gradual increase in the motivation to consume due to increasing tolerance and withdrawal symptoms when the drug is no longer taken [85]. Automatic and habit-related behavior is not affected by the fact that the drug and the reward it procures become increasingly less attractive (conflict).

### 3.2. From Craving to Anxiety, Sleeplessness, and Anhedonia

**Figure 1:**
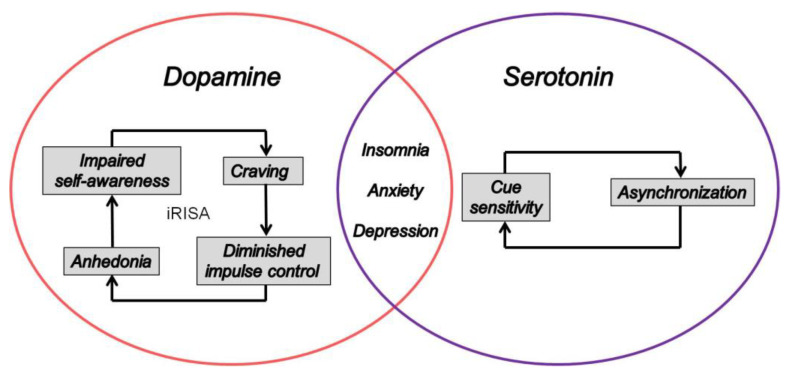
The iRISA syndrome [76] in addiction is centrally controlled by dopamine in the brain, while asynchronization, presumed to be linked to cue sensitivity in digital addiction [50,51,52,53,54], is centrally controlled by serotonin. A deficit in both neurotransmitters is identified as a brain correlate of insomnia, anxiety, and depression.

Chronic drug consumption in addicts leads to adaptations and opposing processes (*allostasis*) producing irritability, *dysphoria*, anxiety, and, finally, *anhedonia* in what is referred to as the brain’s “anti-reward system” [85,86,87]. Drugs usurp everyday behaviors such as eating, sexuality, sports, and others, thereby usurping the natural effect of substances or activities that produce pleasure through the release of dopamine. Acute drug use decreases the reward threshold, while chronic consumption increases this threshold, hence the need to consume more of the drug to reach it [85,86]. The acute effect of the drug increases the concentration of extracellular dopamine through mechanisms including (i) decrease in the inhibitory tone exerted by GABAergic neurons on dopaminergic neurons [85,86,87,88], (ii) release of opioids and endogenous cannabinoids [89], and (iii) a direct action on dopaminergic neurons by increasing their frequency of discharge (firing rate and bursting), with a low-frequency rhythmic oscillation (0.5–1.5 Hz) in the increased firing activity [90]. The released dopamine not only underlies the pleasurable effects but is also involved in much more complex phenomena of attribution of the “added value” (“incentive value”) associated with the drug. A contextual cue associated with the drug, after a period of conditioning, replaces the value of the drug itself and is able to precipitate relapse by an overwhelming, urgent, and irrepressible desire to consume the drug. This has been related to changes in the amygdala [73], resulting in negative emotional states where drug taking becomes an attempt to temporarily alleviate them. In a genuine addict, drug consumption is correlated with attenuated dopamine receptor expression [69,70,71], and the motivation to consume is increased to compensate for the difference between the magnitude of the expected reward and the actual experience. The associated asynchronization of circadian rhythms [50] is centrally controlled by serotonin. Dysfunctional sleep patterns as a consequence of perturbed circadian rhythms have been linked to increased cue sensitivity in digital addicts [50,51,52,53,54]. The motivation to seek the drug, initially potentiated by the dopamine increase during the first intakes, turns into compulsive drug taking against the backdrop of negative emotions in response to the progressively decreasing intensity of the reward. The resulting serotonin-controlled sleep problems reinforce the addictive loop and increase cue sensitivity further (Figure 1). Dopamine release also relays a mechanism where a neutral contextual cue that has been repeatedly matched to drug use is no longer followed by drug delivery [91,92]. Brain imaging studies have shown that, in dependent subjects, supra-physiological levels of dopamine in the NA are associated with a marked decrease in dopaminergic function, including a reduction in dopamine D2 receptor levels [91,93]. Dopamine D2 receptor deficiency could also play a major role in the vulnerability to becoming dependent, as a decrease in dopaminergic transmission is responsible for widespread decrease in the sensitivity of the reward system to the effects of natural rewards [71,72]. The identification of the brain circuitry of reward [94,95] harkens back to the 1950s and had shown, in rats, that the electrical self-stimulation of certain brain structures including the VTA are associated with fatal fasting. This demonstrates that the activation of these brain regions surpasses the activation level induced by natural rewards such as food [95]. Hence, the drug effect substitutes for that of natural rewards, which our brain is programmed for, while a drug has a more intense and prolonged effect [94,95].

## 4. The Dopamine–Serotonin Imbalance Hypothesis

For all addictions, the common denominator is poorly controlled reward-seeking behaviors that lead to functional impairment, distress, depression, insomnia, and, in extreme cases, suicide [96]. Several factors appear to be predictive of digital addiction, including personality traits; the family context; pre-existing use of other drugs such as alcohol, nicotine, or caffeine; and social anxiety [97,98]. Further evidence links the chronic effects of drugs, including the digital drug, to disturbances in the cellular and molecular mechanisms underlying reward and memory processes [99,100,101,102,103].

### 4.1. Synaptic Plasticity and Pathological Adaptation

Even after long periods of abstinence, risk of relapse is precipitated by drug-associated cues, and learning processes have therefore been attributed a major role in the maintenance of addictive behavior [104,105,106]. This supports earlier theory where addiction was described as the pathological usurpation of neural processes that serve normal reward-related learning [105,106]. Several types of neuroadaptation may occur in addiction, including synapse-specific adaptations (synaptic plasticity) underlying specific long-term associative memory [107,108]. The GABAergic and glutamatergic systems [107,108,109], which are privileged targets of alcohol [110,111], are essential players in phenomena of synaptic plasticity [112] and pathological memory [112]. In digital addiction, the underlying control processes are particularly reduced when individuals with internet addiction, for example, are confronted with digital cues representing their preferred use or drug. The interactive functions of the internet [113,114] are considered the most addictive cues here, as they give instant access to other cues, such as online intimacy, and to interaction-specific rewards, such as immediate replies, comments, “likes”, and so forth. Processing such digital cues measurably interferes with working memory performance and general decision making [114]. Other data consistently demonstrate that drug-related characteristics found in behavioral addictions are also important for understanding digital addiction [3,13,14,114,115]. Pathological memory processes explain, at least in part, how drugs leave traces in the brain, which means that even after a very long period of abstinence, the dependent subject can relapse through even the weakest re-use or during exposure to a contextual clue that had been associated with the regular drug use [116]. Recent pre-clinical studies have shown that the transition from controlled cocaine consumption to addiction is linked to the loss of the capacity of NA neurons for the long-term depression of synaptic transmission (i.e., synaptic plasticity) in terms of a lasting decrease in transmission efficiency [117]. Drug-induced synaptic plasticity in several brain regions involved in positive reinforcement have been proposed as the crucial cellular mechanism that ultimately leads to addiction [108]. Persistent changes in behavior, induced by environmental cues (trigger *stimuli*) or by chronic drug use, are most certainly relayed by lasting changes in synaptic transmission and neuronal excitability and even in the number of neural connections. These lasting changes in synaptic transmission, or long-term synaptic plasticity, are generally defined as a change in the efficiency of transmission at a particular synapse [118]. The adaptation of the surface contact points and their characteristics in these synaptic changes is called morphological plasticity. In the state of current knowledge, these plasticity phenomena appear to be the best neurobiological substratum explaining the mechanisms of learning and memorization in addiction [119]. Many studies have demonstrated that strong links between addictive behavior and synaptic plasticity drugs induce persistent changes in communication between certain neurons in the cerebral reward circuits in the cascade of events that leads to addiction [120]. If the drugs increase the efficiency of certain excitatory synapses of dopaminergic neurons of the VTA, this modifies the release of dopamine in target structures to which it sends projections, such as the amygdala and the prefrontal cortex. This ultimately leads to altered (pathological) learning, functionally linked to dopamine [120,121,122,123,124,125,126]. Dopamine and serotonin neurotransmission follow functionally identified pathways in the brain, with interactions involving the amygdala and the hippocampus, which play an important role in drug dependence by mediating the pathological memory mechanisms associated with craving, reward, and withdrawal [119,120,121,122].

### 4.2. Lifestyle-Induced Metabolic Changes

**Figure 2:**
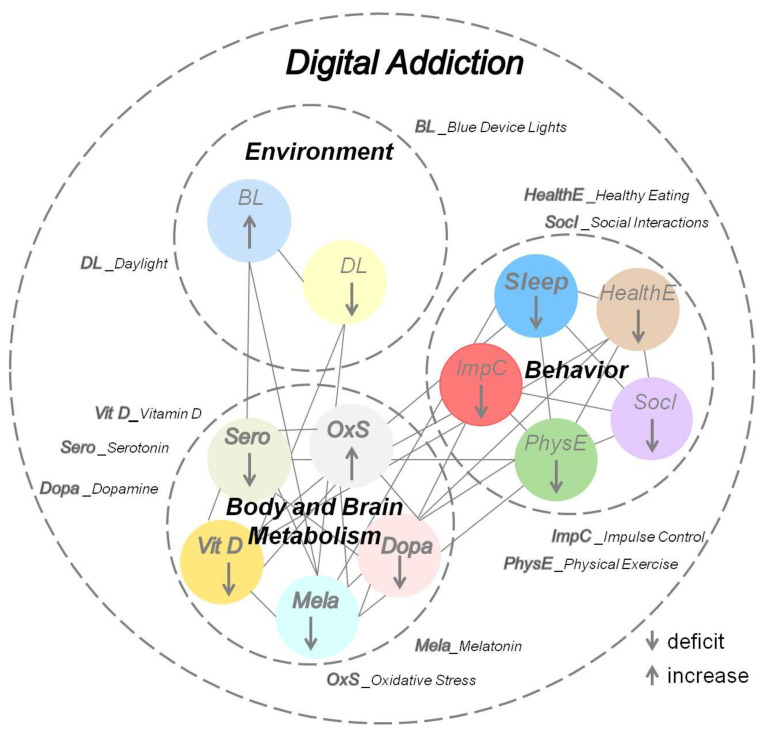
Sleep dysfunction in digital addiction is identified as the key part of a set of mutually reinforcing behaviors. These are the consequences of a perturbed brain–body metabolism, controlled by complex chains of functionally interdependent mechanisms triggered by internal (predisposition, sensitivity) and environmental states.

Addictions often cause individuals to spend more time indoors consuming the drug and long hours of digitally mediated online activities involving sustained exposure to blue device lights for long hours in the day and at night [13]. This behavior has been associated with loss of sleep and symptoms of depression in students [21,27,35,65]. When individuals have to get up early to go to work or college, reduced sleep times or disturbed sleep patterns due to excessive online activities, often combined with a lack of physical exercise, can take a serious toll. Several studies have linked disturbed sleep as a consequence of online activities to poor performance in school, impaired learning, and psychological problems [127,128,129,130]. Electronic media are a source of blue light and, especially when used before bedtime, have a negative impact on individuals’ sleep patterns even when they are not digital addicts [13]. Significant links between digital exposure and lifestyle variables tested in different studies point towards sleep disorders [65] and increased energy intake as the prominent causal link between digital exposure time and obesity [131,132]. Digital addiction produces important metabolic changes, including reduced serotonin availability [133,134,135,136] and perturbed circadian rhythms [28,35] induced by long hours spent online until late at night. The resulting sleep disturbances produce a clinical syndrome referred to as asynchronization [50,137], i.e., disturbance of biological rhythms due to decreased activity of the serotonergic system. The major trigger of asynchronization is hypothesized to be a combination of excessive device light exposure in the night and lack of natural light exposure during daytime [13,137]. Asynchronization in internet abusers was found to be significantly correlated with varying degrees of depression, anxiety, and fatigue [136,137], with vitamin D [138,139] and melatonin [136] deficiency both involved in producing chronic muscular fatigue [140] and disrupted circadian rhythms [136,137]. Therefore, a variety of metabolic factors appears as directly linked to the dopamine and serotonin pathways [138,139,140,141,142] and may contribute to the deficit in availability of both neurotransmitters. Withdrawal from chronic drug use leads to a disruption of regulatory processes in the prefrontal cortex [76,78,80] induced by a decreased synaptic availability of dopamine and serotonin. This double deficiency contributes to withdrawal symptoms, drug craving, and ultimately relapse, as described in the iRISA syndrome [76]. A systematic review focused on fMRI studies involving adult IAD patients free from any comorbid psychiatric condition [143] yielded a total number of 666 tested individuals [143], generating study data acquired during resting state and other paradigms, such as cue-reactivity, guessing, or cognitive control tasks. Most of them were male (95.4%) and young (21–25 years). The most represented internet addiction subtype reported in more than 85% of these patients was the internet gaming disorder or videogame addiction. In resting-state studies, the most relevant abnormalities are localized in the superior temporal gyrus, limbic, medial frontal, and parietal regions. Interestingly, less than half of the papers analyzed in the review [143] reported behavioral differences between patients and normal controls. Yet, all of them found significant differences in cortical and subcortical brain regions involved in cognitive control and reward processing: the orbitofrontal cortex; insula, anterior, and posterior cingulate cortex; temporal and parietal regions; brain stem; and caudate nucleus. This suggests that significant changes occur in the brain that precede observable changes in behavior. A preliminary working model of the specific interactions producing double dopamine–serotonin deficit in the still-developing brains of young digital addicts was proposed recently [13], recognizing that further research is urgently needed for a deeper understanding. Dysfunction of the dopaminergic system is associated with a variety of nervous system diseases. Dopamine levels in the brain and the periphery (blood) increase in response to any type of reward, chemical substance, and/or non-chemical drug [142,144,145,146,147,148]. Dopamine transmitter pathway deregulation is also a well-known consequence of oxidative stress in the body [145]. Functional interactions between the serotonergic and dopaminergic transmitter pathway systems have been identified in association with vitamin D deficiency, stress-induced brain states, and neuropathologies affecting motor and impulse-control function [147,148,149]. In digital addiction, many complex interactions between brain mechanisms underlying compulsive reward seeking and metabolic changes due to lifestyle and environmental context or pre-existing individual (psychological and physiological) factors form a chain of interdependent variables that need to be tackled holistically (Figure 2). Sleep dysfunction does not happen in a void but results from complex interactions between internal (body and brain function) and external (environment) variables. These interactions need to be recognized and fully taken into account for approaching pathological sleep in digital addiction [27,35,43,49,50,51,52,53,54,55,56,57,58,59,60,61,62,64,65,66], as they have implications for preventive measures and clinical treatment.

## 5. Perspectives for Clinical Research and Treatment

The long-term consequences of digital addiction on sleep and the well-being and general health of addicted individuals are yet unknown. Like any compulsive drug use, digital addiction emerges as the result of a loss of prefrontal cortical inhibitory control over drug-seeking habits, as some of the evidence reviewed here has shown. Sensitivity to digital cues forms on the basis of cue reactivity, a learning mechanism in which various cues become associated with the rewarding properties of the drug [150]. Additionally, too little is still known about the social, environmental, neurobiological, and personal factors that determine individual sensitivity to such cues and thereby the likelihood of becoming an addict. As in any addiction, the importance of behavioral impulsivity as a vulnerability trait predicting abuse and addiction [151,152] needs to be addressed. The specific environmental context in which digital addicts evolve, progressively spending more and more time indoors hooked to a connected device, engenders brain and other metabolic disorders, as shown here. Outdoor activities, exercise, and exposure to daylight, which are indispensable for healthy sleep patterns, are replaced by a sedentary lifestyle, bad eating habits, and excessive exposure to blue device lights for long hours at night. Behavioral therapy [153] is a first choice for breaking this complex chain of interdependent factors. Since compulsive behaviors are characterized by executing actions on “autopilot”, mindfulness techniques [154] were found effective in inducing individuals to relate more consciously to their environment and help them snap out of the compulsive loop. Furthermore, mindfulness techniques have proven effective for reducing insomnia-related arousal [155] and therefore could help digital addicts recover restoring sleep patterns, with direct benefits for their general metabolism, which then could facilitate their return to a healthier general lifestyle. To reduce drug seeking, promote abstinence, and prevent relapse, pharmacological treatment may need to be envisaged in digital addicts with severe insomnia. This would imply resorting to chemicals that directly target the serotonergic and dopaminergic neurotransmitter systems similar to those currently used to treat depression and schizophrenia [142]. As some of the evidence reviewed here has shown, these neurotransmitter systems are critically important to the functional integrity of reward mechanisms, sleep patterns, and mood. If these can be stabilized in a digital addict, the power of addictive cues such as interactive functions on the internet and their associated rewards could lose some of their power and help individuals get “unhooked”. Some novel brain stimulation therapies for depression and schizophrenia may complement or assist the clinical treatment of *digital addiction*. Examples here would be VNS (Vague Nerve Stimulation) and TMS (Trancranial Magnetic Stimulation), which have proven to a given extent successful in treating depression [156]. VNS produces good results in severe cases of treatment-resistant depression (TRD), while TMS has demonstrated efficacy on milder forms of TRD. Deep brain stimulation (DBS), previously used successfully for the treatment of movement disorders, has been extended to the treatment of psychiatric disorders such as depression and schizophrenia [157]. DBS involves high-frequency stimulation of deep brain areas through electrodes implanted under stereotactic surgery. It produces functional inhibition in the region around the electrode [158] and excitatory effects on local axons and more distant loci. In the longer term, it corrects pathological brain activity in brain networks with connections to the implantation site [159]. Unlike other forms of neurosurgery, DBS is reversible: the stimulation can be turned off and the electrodes explanted without any permanent loss of function. Although DBS is still an emerging treatment, promising efficacy and safety have been demonstrated in preliminary trials in patients with treatment-resistant depression (TRD). Neuroimaging has permitted to identify DBS targets such as the dopaminergic system controlling reward behaviors and the serotonergic system ensuring mood control [160]. The NA comprises most of the ventral striatal DBS target, and focal DBS of the NA positively influences reward-seeking behavior and anhedonia in the syndrome of depression [161,162,163]. Positive symptoms of schizophrenia, i.e., delusions and hallucinations, are linked to ventral striatal overactivity with increased and inappropriate dopamine activity and give rise to abnormal reward prediction error signals. This suggests ventral striatal electrode placement for the treatment of schizophrenia [164] and obsessive compulsory disorder [165]. The basal ganglia (BG) are a choice target for DBS, and both direct and indirect BG pathways are modulated by dopaminergic neurons [166]. The BG receive major afferent input from the hippocampus. The ventral striatum is involved in the control of the dopaminergic nigro-striatal pathway, while the *caudate nucleus* influences the hippocampal theta rhythm and inhibits hippocampal spike activities [167]. This may open perspectives for the treatment of pathological memory processes in addiction, including *digital addiction*. In addition, there is now growing evidence that binaural-beat exposure may be a choice for minimally invasive, effective therapy of anxiety syndromes and sleep disorders [168,169,170], including those associated with internet addiction disorder [171,172]. The therapy can be administered without prior training and consists of presenting two tones with different sound frequencies separately but simultaneously to each ear. The selective choice of specific frequencies modulates activity in brain areas affected by addiction and their connectivity [173,174]. The direction and the magnitude of measurable therapeutic effects on cognitive function and behavior patterns depends upon the sound frequency, duration of exposure, and the context in which exposure takes place [175,176]. Structural and functional brain mechanisms underlying digital addiction correlated with sleep loss have been identified in functional magnetic resonance imaging studies, as extensively reviewed in [177]. Adolescents and adults diagnosed as digital addicts had reduced gray matter volume in regions associated with attention motor coordination executive function and perception. Adolescents showed lower white matter (WM) integrity measures in several brain regions involved in decision making, behavioral inhibition, and emotional regulation. They showed disruption in the functional connectivity in areas responsible for learning, memory, and executive function and the processing of auditory, visual, and somatosensory stimuli relayed with motor signals. This picture is accompanied by a decreased functional connectivity of the parieto-frontal cortical (striatal) circuitry and an increased functional connectivity of several executive control regions functionally related to depression.

## 6. Conclusions

Teenagers today sleep less than previous generations, connected in a digital world driven by social demands for immediacy and engendering chronic sleep deprivation [178]. Analysis of some of the most recent pertaining literature here has shown that a functional imbalance in the dopamine–serotonin neurotransmitter pathways provides a consistent account for a silent process that leads from increasingly compulsive behavior to global functional impairment accompanied by sleeplessness or insomnia, emotional distress, depression, and sometimes suicide. Sleep dysfunction does not happen in a void but results from complex interactions between body, brain, and the environment. In digital addiction, it is a critical symptom within intertwined chains of causes and effects that are difficult to disentangle (Figure 2). The number of smartphone users worldwide today surpasses six billion, and it is forecasted to further grow by several hundred million in the next few years [179]. Although China, India, and the United States are the countries with the largest number of smart phone users and are also the countries where most of the research on digital addiction has been carried out up to now, we can expect similar trends all over Europe in the not-so-distant future. This overview makes clear why digital devices, by providing anyone with internet access anywhere anytime, deliver the “new drug” of the 21st century. This has produced a new form of worldwide sleep disorder that represents a severe public health issue. Essential brain mechanisms for healthy motivation, memory, eating, mood, and a good night’s sleep are now deregulated in large populations of increasingly younger individuals, including toddlers [180], worldwide. Whether some internet activities may be more addictive or detrimental to good sleep and a healthy lifestyle than others is an open question [181]. Increases in digital addiction during the COVID-19 pandemic, related to financial hardship, bereavement, isolation, anxiety, and stress, were reported in studies from different countries [66,182,183,184]. This further prompts our awareness towards recognizing a new worldwide syndrome fueled by increasingly adverse societal conditions. This syndrome needs to be approached holistically. Educational measures are urgently needed to prevent further damage to the youngest members of society. Clinical research should aim for effective, holistic strategies for therapy. Sleep is severely disturbed in all digital addicts. A premium could therefore be placed on improving sleep quality in these patients, as sleep is the key to giving the brain the rest it needs to “snap out” of the compulsory loop.

## Data Availability

All data supporting this analysis can be found in the references cited.
